# Complications of Dermal Injectables—A Retrospective Study

**DOI:** 10.1111/jocd.70582

**Published:** 2026-01-19

**Authors:** Lynhda Nguyen, Stefan W. Schneider, Ute Siemann‐Harms, Katharina Herberger

**Affiliations:** ^1^ Department of Laser and Aesthetics, Department of Dermatology and Venereology University Medical‐Center Hamburg‐Eppendorf Hamburg Germany; ^2^ Department of Dermatology and Venereology University Medical‐Center Hamburg‐Eppendorf Hamburg Germany

**Keywords:** aesthetic medicine, filler complications, injectables, vascular occlusion

## Abstract

**Background:**

Hyaluronic acid (HA)‐based injectables are generally considered safe. However, short‐ and long‐term complications may occur.

**Objective:**

To evaluate complications related to soft‐tissue augmentation presented to the Department of Dermatology at a university hospital.

**Materials and Methods:**

A retrospective analysis was conducted using electronic medical records from January 2019 to January 2025. Adverse events related to HA‐based injectables, their management, and outcomes were identified and evaluated.

**Results:**

Thirty‐one patients (mean age: 50.4 years) were included in the analysis. Injections have been administered by cosmeticians (2/31), nonmedical practitioners (2/31), or individuals of unknown qualification. The most common complications were biofilm formation and chronic immune reactions, followed by abscesses, Tyndall effect, vascular occlusion, and filler migration. Over half of the products involved were either uncertified or of unknown origin.

**Conclusion:**

The findings highlight the need for qualified practitioners, careful product selection, and adherence to standardized techniques. Enhancing practitioner education, establishing evidence‐based protocols, and strengthening regulatory oversight are essential to ensure patient safety in aesthetic medicine.

## 1 Introduction

Dermal injectables have become increasingly popular as a nonsurgical treatment option for addressing aging skin and restoring lost volume. According to recent data from the *American Society of Plastic Surgeons*, nonsurgical cosmetic procedures have surged by approximately 50% between 2019 and 2022, totaling 23.7 million procedures in 2022 alone [1]. Among these, filler injections have seen an increase of about 70%, with 4.9 million procedures performed in 2022 [1]. Hyaluronic acid (HA)‐based fillers dominate this category, widely used for smoothing fine lines, enhancing contours, and replenishing facial volume [1, 2].

These treatments are widely regarded as having a high safety profile, and adverse effects (AEs) are generally rare [3, 4, 5]. However, incomplete patient history, improper techniques, inadequate adherence to best practices, or use of noncertified products can result in severe long‐term complications [6]. Therefore, consensus recommendations and clinical practice guidelines have been published to support healthcare professionals in diagnosing, preventing, and managing complications related to injectables [7, 8].

This study aims to examine complications arising from soft‐tissue augmentation presented at a university hospital. By analyzing the incidence, management strategies, and outcomes of these AEs, the present study seeks to provide insights into improving patient safety.

## 2 Materials and Methods

### 2.1 Study Design

A retrospective chart analysis was performed using the electronic patient file software (Soarian, Siemens) from January 2019 up until January 2025. We analyzed data of patients who consulted the Department of Dermatology at the University Medical Center Hamburg‐Eppendorf due to different complications following injectable treatments performed outside the clinic.

### 2.2 Data Collection

We reviewed data including age, gender, type of injectable, type of practitioner who performed the injection, time to occurrence of AEs, symptoms and treatment of AEs, as well as outcome.

### 2.3 Statistical Analysis

Statistical data were performed using SPSS (IBM, Version 29.0.2.0). Descriptive data were used to report the demographic data of patients. Data were presented as means ± standard deviation and ranges (minimum–maximum).

## 3 Results

### 3.1 Basic Characteristics

A total of 31 patients were included in the analysis, with a mean age of 50.4 ± 12.3 years (range: 25–75). The majority of patients (96.8%) were female. Reported complications varied but were broadly categorized based on the time of onset into vascular occlusion and tissue necrosis, infections, Tyndall effect, filler migration, and biofilm‐related or chronic immunological reactions. The most frequently treated area was the cheeks (26.5%), followed by the under‐eye region (20.6%) and the lips (14.7%). A detailed overview of patient characteristics is provided in Table 1.

**TABLE 1:** Patient characteristics.

Characteristics	*n* (%)
Male/female	1/30
Age (mean ± SD (minimum–maximum))	50.4 ± 12.3 (25–75)
Treated areas	
Cheeks	9 (26.5)
Under‐eye area	7 (20.6)
Lips	5 (14.7)
Nasolabial folds	4 (11.8)
Nose	3 (8.8)
Chin	3 (8.8)
Forehead	1 (2.9)
Glabella	1 (2.9)
Knee	1 (2.9)
Symptoms	
Vascular occlusion and tissue necrosis	4 (12.9)
Erysipelas	5 (16.1)
Abscess	2 (6.5)
Tyndall effect	7 (22.6)
Migration	3 (9.7)
Late‐onset nodules	10 (32.1)
Used products	
FDA‐certified hyaluronic acid‐based filler	15 (48.4)
Not‐certified hyaluronic acid‐based filler	4 (12.9)
Unknown	12 (38.7)
Practitioners	
Nonmedical practitioner	4 (13.0)
Dermatologists	4 (12.9)
Plastic surgeons	1 (3.2)
Family doctor	1 (3.2)
Medical microbiologist	1 (3.2)
Unknown	20 (64.5)

### 3.2 Immediately After Injection

#### 3.2.1 Vascular Occlusion and Tissue Necrosis

**FIGURE 1:**
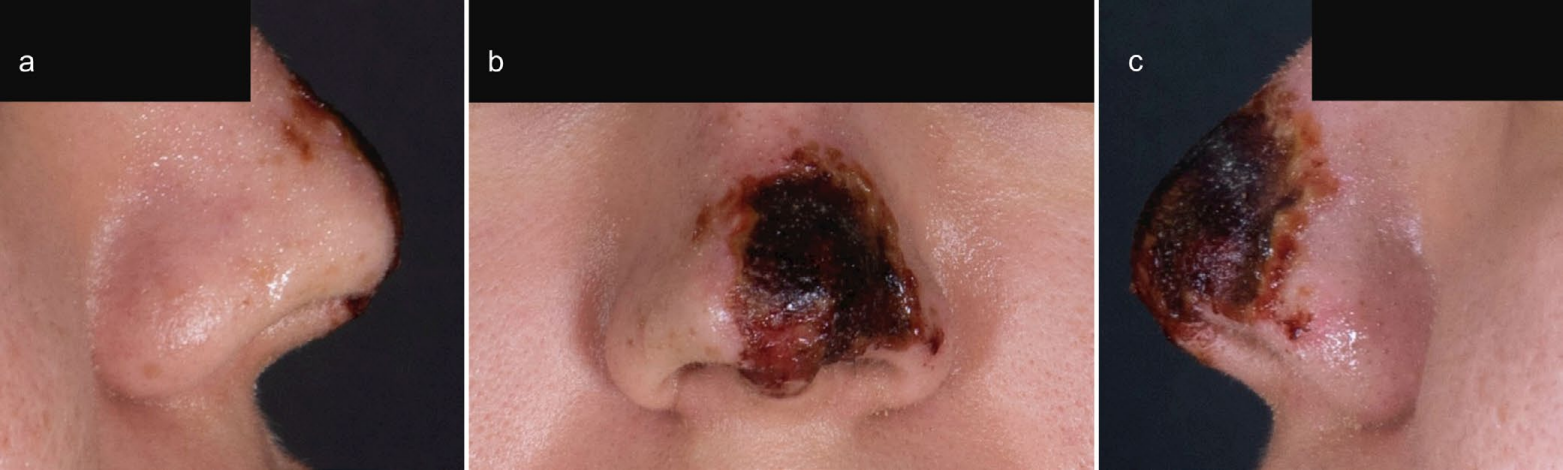
Patient presenting with nasal skin necrosis, shown from the right (a), frontal (b), and left (c) side.

We assessed four patients (4/31; 12.9%) who presented with vascular occlusions following treatment, three of whom progressed to skin necrosis (Figure 1). Two patients experienced complications in the nasal region, one on the forehead, and one on the knee. Each patient reported sudden, sharp pain at the treatment site, followed by the development of a livid erythematous discoloration. To address the occlusions, all patients received injections of hyaluronidase over consecutive days. Additionally, comprehensive wound management with antiseptic wound dressings was administered. Close follow‐up visits were scheduled to assess tissue viability.

### 3.3 First Days up to Few Weeks

#### 3.3.1 Infection

We evaluated seven patients (7/31; 22.6%) who developed painful swelling and nodules within a few days following injectable treatment. Despite experiencing localized inflammatory symptoms, none of the patients exhibited fever or systemic deterioration. After being treated by their HA filler providers, all patients had received hyaluronidase treatment from their respective dermatology practices before presenting at our clinic, which provided only mild symptom relief. Additionally, one patient had been prescribed systemic prednisone. Upon further assessment, we treated these patients with systemic antibiotics. In two cases where significant abscess formation or persistent swelling was observed, incision and drainage were performed to facilitate resolution. The abscess material was sent for microbiological culture, which yielded coagulase‐negative *Staphylococcus*.

### 3.4 Few Weeks to Months

#### 3.4.1 Tyndall Effect

We evaluated seven patients (7/31; 22.6%) who presented with a Tyndall effect and lymphatic edema in the under‐eye region several weeks to months after receiving HA‐based filler injection. The Tyndall effect, characterized by a blue‐grayish discoloration of the skin due to superficial placement of HA filler, was the primary aesthetic concern. Additionally, patients exhibited localized swelling, but none reported pain, erythema, or other signs of infection. To address these complications, we administered a hyaluronidase injection. Following treatment, patients experienced significant improvement.

#### 3.4.2 Migration

Three patients (3/31; 9.7%) presented at our department due to migration of HA filler. Two patients experienced filler migration after lip augmentation. The third patient had HA injected into the cheeks, which subsequently migrated into the enoral region. To address the issue, all three patients were treated with hyaluronidase injections. They reported relief from the undesired effect.

#### 3.4.3 Delayed‐Onset Reaction: Late‐Onset Nodules

**FIGURE 2:**
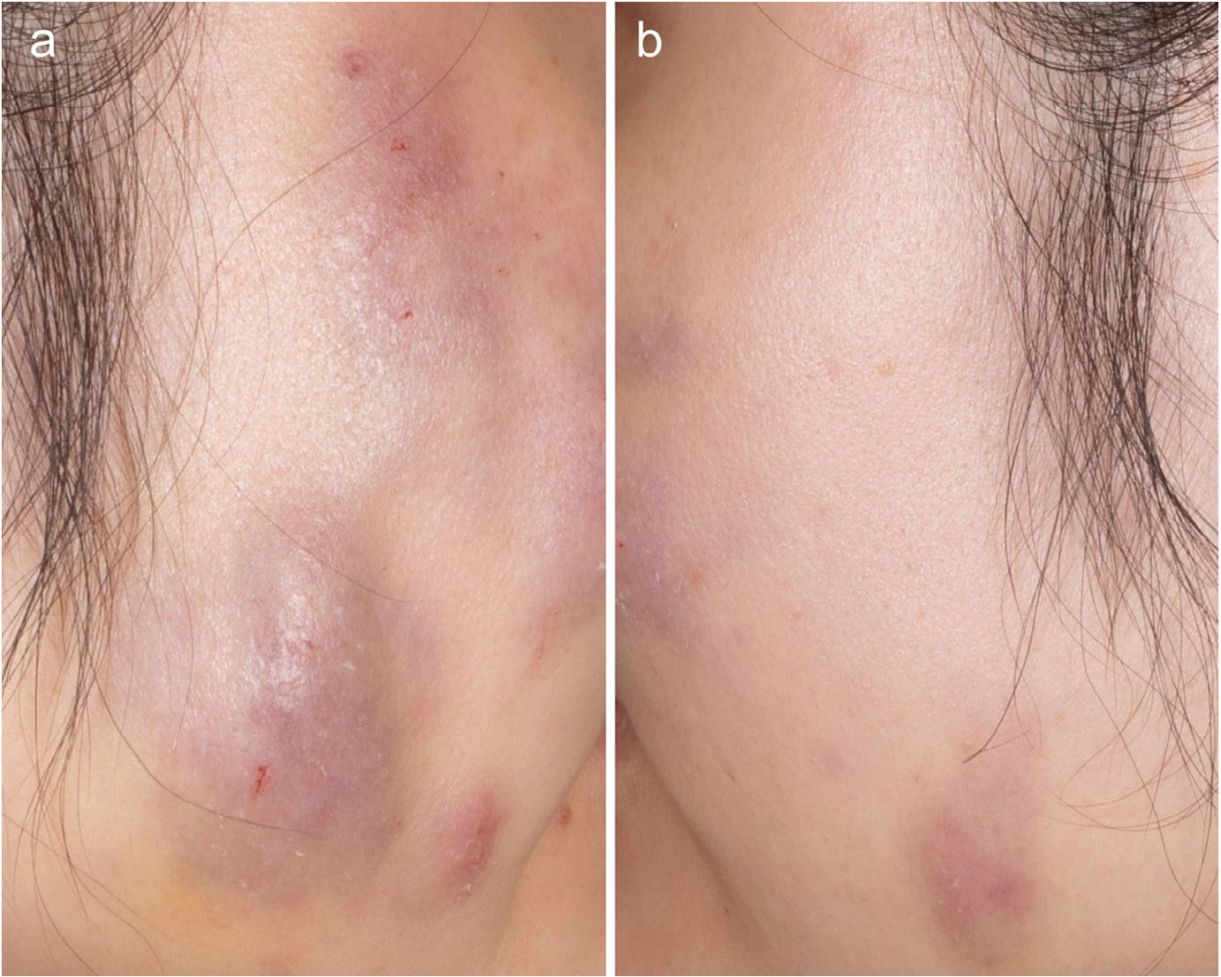
Patient presenting with multiple nodules on both cheeks. Shown from the right (a) and left (b) side.

Ten patients (10/31; 32.1%) presented to our department with intermittent swelling, erythema, and formation of multiple nodules occurring several months to years after receiving injectable filler treatments (Figure 2). Upon reviewing their histories, only half of the patients had received FDA‐approved filler products. Notably, two patients were unable to recall any previous filler injections. For diagnostic evaluation, biopsies were performed, confirming findings consistent with a foreign body reaction with HA. In one case, where a patient had previously received filler injections in the cheeks, magnetic resonance imaging was conducted to assess the extent of involvement and to rule out deeper tissue complications. Triggering events like dental procedures, local or systemic infections at the time of injections, could not be ruled out completely by the patients after such a long time.

Initial treatment consisted of systemic antibiotics to address potential bacterial involvement and target biofilm formation. Subsequently, hyaluronidase was administered into the affected nodules. In one case of persistent nodules unresponsive to conservative treatment, surgical excision was performed to remove the affected tissue.

## 4 Discussion

This single‐center study analyzed filler‐related AEs in 31 patients over recent years. With the rising popularity of HA‐based injectables in aesthetic medicine, there is also an increasing awareness of potential AEs.

According to existing literature and expert consensus, filler‐related complications can be classified based on their time of onset and severity, ranging from localized to systemic reactions [7, 9]. In our cohort, the majority of patients experienced complications arising in a short time post‐treatment, including vascular occlusion, bacterial infections, Tyndall effect, and filler migration. In addition, approximately one‐third of patients presented with long‐term complications suggestive of biofilm formation and chronic inflammatory reactions.

In around half of our cases, the filler product used was not FDA‐certified or unknown, making the planning of an appropriate treatment difficult. This highlights the importance of product choice in complication prevention. When FDA‐approved HA‐based fillers are used, physicians must be familiar with their distinct rheological properties in order to select the most appropriate product for the treatment area, the desired aesthetic outcome, and the patient's individual characteristics [10]. A wide range of fillers with different profiles is available, and a thorough understanding of these differences is essential for safe and effective application. Additionally, the market for dermal fillers continues to expand. Given this growing landscape, it is crucial for practitioners to prioritize the selection of fillers from reputable companies that adhere to the highest manufacturing standards. Ideally, these products should undergo rigorous testing in clinical studies to demonstrate their safety, efficacy, and longevity. Using well‐established, high‐quality fillers not only enhances treatment outcomes but also minimizes the risk of complications.

Another key issue contributing to filler‐related complications is the current regulatory framework surrounding soft tissue fillers. Currently, soft tissue fillers are classified as medical products rather than pharmaceuticals. This classification results in a less stringent approval process and regulatory oversight. Moreover, there are inconsistencies regarding who is legally permitted to perform injectable procedures in Germany. For example, non‐medical practitioners (German: Heilpraktiker:innen) are legally permitted to administer injectables, despite potentially having limited knowledge of facial anatomy, sterile techniques, risk factors, or complication management. Additionally, we encountered cases where cosmeticians performed filler injections, despite not being legally authorized to do so in Germany. The administration of fillers by individuals without adequate medical training can lead to suboptimal aesthetic outcomes, higher complication rates, and delayed or improper management of AEs.

The number of Tyndall effects highlights the importance of appropriate filler placement, particularly in delicate areas such as the periorbital region [11, 12, 13]. HA migration is a known complication that can occur due to various factors, including injection technique, filler properties, and individual tissue response [14]. While it does not always cause functional impairment, it can lead to aesthetic concerns and patient dissatisfaction.

The cases presented in the study underscore the importance of promptly recognizing and managing short‐ and delayed‐onset inflammatory reactions following injectable treatments. While hyaluronidase can aid in symptom reduction, it may not be sufficient in cases involving bacterial infection or abscess formation. A multidisciplinary approach incorporating antibiotics, targeted hyaluronidase, and, when necessary, surgical intervention, is essential.

This study has some limitations. First, its retrospective nature introduces the potential for selection bias. Second, as a monocentric study, the findings may not be generalizable to all clinical settings or patient populations. Additionally, some key information, such as the specific filler material used in certain cases, was unavailable, as many patients were unsure of the exact product injected. This lack of documentation complicates the assessment of product‐specific AEs.

This study provides valuable insights into filler‐related complications observed in patients after being treated outside of our department. By analyzing the incidence, presentation, and management of these complications, we aim to contribute to the ongoing effort to enhance patient safety and treatment outcomes in aesthetic medicine. Our findings underscore the importance of proper practitioner training, careful product selection, and adherence to standardized injection techniques. Additionally, a notable proportion of complications occurred following treatments in the under‐eye area, raising the question of whether this region represents an appropriate and justifiable indication for injectable procedures. Furthermore, regulatory measures should be strengthened to ensure that only qualified medical professionals perform injectable treatments. In addition, implementing a complication reporting system could be beneficial, both for the early identification of AEs and for providing appropriate support in their management. Ultimately, improving education, establishing evidence‐based protocols, and promoting stricter regulatory oversight will be key to ensuring the highest standards of patient care in the field of aesthetic medicine.

## Author Contributions

L.N.: Data curation, data interpretation; writing; S.W.S.: Data interpretation; U.S.‐H.: Data curation, supervision; K.H.: Data curation, supervision.

## Ethics Statement

The patients in this manuscript have given informed consent to the publication of their case details.

## Conflicts of Interest

Lynhda Nguyen and Katharina Herberger have received lecture fees from Cynosure Lutronic. Stefan W. Schneider and Ute Siemann‐Harms have none to be declared.

## Data Availability

The data that support the findings of this study are available from the corresponding author upon reasonable request.
